# Hydropriming and Biopriming Improve *Medicago truncatula* Seed Germination and Upregulate DNA Repair and Antioxidant Genes

**DOI:** 10.3390/genes11030242

**Published:** 2020-02-25

**Authors:** Chiara Forti, Ajay Shankar, Anjali Singh, Alma Balestrazzi, Vishal Prasad, Anca Macovei

**Affiliations:** 1 Department of Biology and Biotechnology ‘L. Spallanzani’, University of Pavia, via Ferrata 9, 27100 Pavia, Italy; chiara.forti01@universitadipavia.it (C.F.); alma.balestrazzi@unipv.it (A.B.); 2 Institute of Environment and Sustainable Development, Banaras Hindu University, 221005 Varanasi, India; shankarajay17@gmail.com (A.S.); singh.anjali241@gmail.com (A.S.); vp.iesd@bhu.ac.in (V.P.)

**Keywords:** abiotic stress, biopriming, *Bacillus* spp., hydropriming, *Medicago truncatula*, qRT-PCR, seed germination

## Abstract

Seed germination is a critical parameter for the successful development of sustainable agricultural practices. While seed germination is impaired by environmental constraints emerging from the climate change scenario, several types of simple procedures, known as priming, can be used to enhance it. Seed priming is defined as the process of regulating seed germination by managing a series of parameters during the initial stages of germination. Hydropriming is a highly accessible and economic technique that involves soaking of seeds in water followed by drying. Biopriming refers to the inoculation of seeds with beneficial microorganism. The present study aims to investigate whether hydropriming and biopriming could enhance seed germination. Thereby, the germination of *Medicago truncatula* seeds exposed to hydropriming and/or *Bacillus* spp. isolates was monitored for two-weeks. The seeds were sown in trays containing two types of in situ agricultural soils collected from Northern India (Karsara, Varanasi). This region is believed to be contaminated by solid waste from a nearby power plant. Phenotypic parameters had been monitored and compared to find the most appropriate combination of treatments. Additionally, *q*RT-PCR was used to evaluate the expression levels of specific genes used as molecular indicators of seed quality. The results show that, while hydropriming significantly enhanced seed germination percentage, biopriming resulted in improved seedling development, represented by increased biomass rather than seedling length. At a molecular level, this is reflected by the upregulation of genes involved in DNA damage repair and antioxidant defence. In conclusion, hydropriming and biopriming are efficient to improve seed germination and seedling establishment in soils collected from damaged sites of Northern India; this is reflected by morphological parameters and molecular hallmarks of seed quality.

## 1. Introduction

Seed germination is a critical parameter for the successful development of both cultivated crops and wild species. While seed germination is impaired by certain conditions (soil contamination, extreme temperatures, pathogens), several types of simple procedures known as priming can be used to enhance it. Seed priming is defined as the process of regulating seed germination by managing parameters like temperature or seed moisture content during the initial stages of germination [1]. By doing so, the priming techniques bring the seed closer to the point of germination. During priming, seeds are advanced to an equal stage of the germination process to enable fast and uniform emergence when planted. Depending on plant species, seed morphology and physiology, different priming treatments can be applied, triggering the so-called “pre-germinative metabolism” [2]. This is characterized by rapid water uptake, which in turn activates a series of dynamic biochemical events such as the use of reserve storage compounds (lipids, carbohydrates, proteins), production of reactive oxygen species (ROS) and subsequent activation of antioxidant enzymes, DNA damage sensing and repair [3]. Indeed, intense DNA repair, essential for de novo DNA synthesis in embryo cells [4], and enhanced antioxidant functions are the prerequisites of a successful germination process and can be used as molecular markers of seed vigour [5,6,7,8,9].

Priming techniques are usually applied during the first phase of germination and are dedicated to accelerate and uniformize the progression of seed through the germination phases. Several seed priming techniques are being currently used; these include treatments with physical (irradiation, ultrasound, magnetic files), chemical (salts, phytohormones, aptamers), or biological (bacteria, fungi) agents [2,10]. Among these, hydropriming is a technique for initiating germination (without the emergence of the radicle) that involves soaking of seeds in water followed by drying. Hydropriming allows the seeds to quickly reach a high level of moisture with a constant supply of oxygen, thus increasing the level of metabolites associated with the germination process. This form of hydration followed by seed drying is highly accessible and economical. Seed hydration treatments have proven to be successful and effective in germinating seeds under a variety of stress conditions [11,12,13,14,15]. Biopriming, on the other hand, involves the use of beneficial soil microorganisms that can act as biofertilizers and promote seedling growth by synthesizing plant hormones or increasing nutrients uptake from the soil [16,17,18,19]. Hence, an interesting aspect of seed priming relates to the boost of seed germination and seedling establishment under non-optimal conditions. Several studies reported that seed priming can alleviate stress levels in seedlings and this can result in better productivity levels under environmental constraints [13,19,20,21,22].

Soil contamination poses a worrisome threat to agricultural productivity, food safety, and human health. It refers to the presence of chemical substances at higher than normal concentrations that have adverse effects on living organisms. The Status of the World’s Soil Resources Report (SWSR) identified soil contamination as one of the main threats affecting global and agricultural ecosystems [23]. This is also the particular case of India as a developing country where industrialization has become heavily intensified and the contamination of agricultural soils has increased substantially [24,25,26]. Contamination of agricultural lands is a major concern for agriculture. Contaminants can be easily taken up by plant roots and translocated into aerial parts, resulting in inhibition of plant growth and, in extreme cases, plant death [27,28]. Moreover, this can also threaten the safety of food chain production as products containing high amounts of contaminants are dangerous to human health [23].

The main objective of the current study was to evaluate the efficacy of hydropriming and biopriming techniques to enhance seed germination, using *Medicago truncatula* as model legume species. Primed and non-primed seeds were grown in trays containing two different agricultural soils collected from Northern India (Karsara, Varanasi). The efficacy on the treatments was evaluated based on phenotypic (germination parameters and seedling biometric measurements) and molecular (expression profiles of genes involved in DNA repair and antioxidant response) aspects. Investigating the expression profiles of genes that can be used as indicators of seed quality and seeding growth [5,6,7,8] is intended to speed up the selection of high-quality seed lots. For instance, *OGG1* (8-Oxoguanine glycosylase) and *FPG* (Formamidopyrimidine-DNA glycosylase) genes, playing similar roles within the BER (Base Excision Repair) pathway, were previously shown to be upregulated during seed imbibition in *M. truncatula* [29]. Similarly, *SOD* (Superoxide dismutase) and *APX* (Ascorbate peroxidase), known for their free radical scavenging activity, are required to balance the production of ROS during seed imbibition [5]. *Medicago* species are mainly used as forage crops and the presence of symbiosis with nitrogen-fixing bacteria can further aid to reduce soil degradation. India has a large number of livestock and the quality of greed fodder is reported to be problematic [30]. Investigating *Medicago* spp. can be of support to expand the knowledge and reduce the existing gap relative to this aspect [30]. Moreover, improving seed germination on local agricultural soils using easy and economically accessible methods will upgrade the livelihood of many small farmers.

## 2. Materials and Methods

### 2.1. Plant Material and Experimental Design

The *Medicago truncatula* (Jemalong commercial variety) seeds used in this study were kindly supplied by Fertiprado L.d.a (Vaiamonte, Portugal). The seeds were subjected to hydropriming and biopriming and grown in trays containing two types of agricultural soils for a time period of two weeks. The soils, herein denominated as Soil_A and Soil_B, were collected from Northern India (namely Karsara, Uttar Pradesh, geographic coordinates 19°38’0” North, 73°29’0” East). Both soil types were regionally collected in situ from the field and no additional treatments were implemented. At the time of soil collection, the land was barren and no sign of recent tillage or any type of fertilization was observed. The chemical characteristics of the two types of soils are presented in Table 1. The two types of soil mainly differ in their content of total dissolved solids (TDS) based on their vicinity to the power plant at Karsara; Soil_B is located closer to the power plant while Soil_A is located in the vicinity of the village. As the first true trifoliate leaves start to appear after the 7th day, the 14th day after sowing was selected to have fully developed seedlings in all treatments.

**Figure 1:**
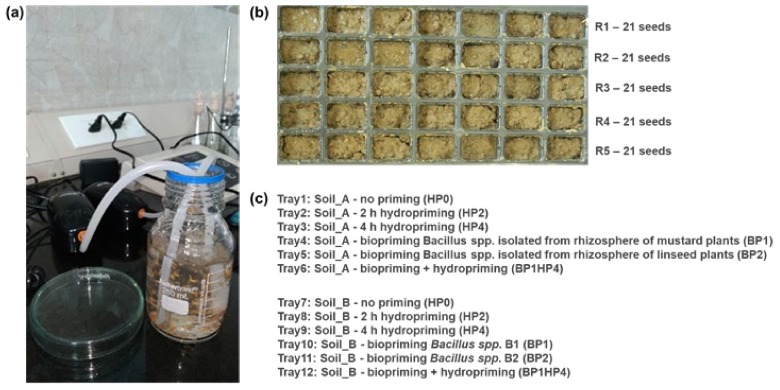
Experimental design. (**a**) Hydropriming treatment of *Medicago truncatula* seeds. (**b**) Germination tray; each row (R1–R5) is considered as a replicate of 21 seeds each. (**c**) Treatment labels.

Hydropriming was carried out in a bottle containing purified water under continuous aeration for 2 h (HP2) and 4 h (HP4) (Figure 1a). The aeration system was constituted by a Wave Air Pump Mouse 2 β aerator (De Jong Marinelife B.V., Spijk, The Netherlands) setup with the following parameters: 220–240 V, 50 Hz, 2.3 W, output 1.8 L min^–1^, pressure 0.012 MPa. A non-primed control (HP0) was also used.

Biopriming was performed with two formulations of bacterial cultures prepared by mixing 1 × 10^8^ cells with 2 g of talc (CDH, New Delhi, India) and jaggery syrup, a standard procedure that helps the adhesion of bacteria to the seed surface. Each bacterial formulation contained a different strain of *Bacillus* spp. The first strain (denominated BP1) was isolated from the rhizosphere of mustard (*Brassica juncea*) plants while the second strain (BP2) was isolated from the rhizosphere of linseed (*Linum usitatissimum*) plants. Both strains tested positive for phosphate solubilization, production of indoleacetic acid and siderophores (Prasad, personal communication). An additional treatment was performed by combining 4 h of hydropriming and the BP1 strain (hereby denominated as BP1HP4). This combination was chosen based on an ongoing characterization of BP1 strain and previous observations of molecular events during seed imbibition [5,29].

Control and primed seeds were sown in germination trays (Figure 1b) kept outside in the open area of IESD, BHU, Varanasi, to simulate local climatic conditions. Trays were watered every three days with 2 mL of water/tray square so that no excess or lack of water would contribute the outcome of the experiment. The experiment was performed in October 2018, when day/night temperature ranged between 28–31/20–24 °C and the relative humidity was around 83%. In a tray, each row was considered as a replicate (R1–R5) containing 21 seeds (three seeds per square).

### 2.2. Phenotypic Analyses

Germination tests were carried out in germination trays containing 21 seeds/row; all experiments were carried out in five replicates. The germinated seeds were counted every 24 h for 14 days. A seed was considered as germinated when the cotyledons were visible above ground. The total percentage (%) of germination was calculated at the end of the experiment, while the time required for 50% of seeds to germinate (T_50_) was calculated according to the formula (1) [31]:

T_50_ = t_i_ + [(N/2 − n_i_) (t_i_ − t_j_)]/n_i_ − n_j_ (1)

where N is the final number of germination and n_i_, n_j_ cumulative number of seeds germinated by adjacent counts at times t_i_ and t_j_ when n_i_ < N/2 < n_j_.

The seedlings length (mm) was measured on millimetric paper at the 14th day. For biomass analysis, seedlings were collected at 14 days after sowing and their fresh weight (FW) was measured (g). Seedlings were then placed in the oven at 60 °C for 24 h, and subsequently, the dry weight (DW) was measured (g). Data are presented as mean ± standard deviation (SD) of five biological and five technical replicates.

### 2.3. RNA Extraction, cDNA Synthesis, and qRT-PCR Analysis

RNA was isolated from primed and non-primed *M. truncatula* seeds and whole seedlings grown in the two types of soils by using the Trizol (Thermo Fisher, Bengaluru, India) reagent, as indicated by the supplier. Total RNA was quantified by agarose gel electrophoresis and spectrophotometric analysis using a WPA Biowave DNA (Biochrom, Cambridge, UK). One microgram of RNA was reverse-transcribed using the RevertAid First Strand cDNA Synthesis Kit (Thermo Fisher, Milan, Italy), while *q*RT-PCR was carried out using the Maxima SYBR Green qPCR Master Mix (Thermo Fisher, Italy) following the suppliers’ instructions. C_t_ values and *q*RT-PCR efficiency, obtained from the Rotor-Gene 6000 Series Software 1.7 (Corbett Robotics, Brisbane, Australia), were analyzed using the REST2009 Software V2.0.13 (Qiagen GmbH, Germany). The *q*RT-PCR was carried out in a final volume of 12 µL using a Rotor-Gene 6000 PCR apparatus (Corbett Robotics, Australia). The amplification conditions were: Denaturation at 95 °C for 10 min (one cycle), followed by 45 cycles of 95 °C for 15 s, 60 °C for 60 s, each. For each oligonucleotide set, a no-template control was used. The *ELF1α* and *GADPH* genes were used as references, as they resulted the most stable under the tested conditions following the geNorm (https://genorm.cmgg.be/) [32] analysis (Supplementary Figure S1). The gene-specific oligonucleotide primers, designed using Primer3 (http://primer3.ut.ee/) [33], are listed in Supplementary Table S1. The PfaffI method [34] was used for the relative quantification of transcript accumulation. Data is represented as relative expression, obtained by dividing (T/R) the expression value of the respective target genes (T) to the expression value of the reference gene (R), obtained as geometrical mean of *ELF1α* and *GADPH* values. All reactions were performed in triplicate and results represented as mean ± SD of each sample.

### 2.4. Statistical Analyses

For each variable (soil and priming), significant differences between treatments were determined with two-way ANOVA (Analysis of Variance) using the statistical tool developed by Assaad et al. [35]. For each treatment, five biological replicates were considered. Means were compared using the Duncan test, where means with a significance value lower than 0.05 were considered statistically different. Letters are used to indicate significant differences among all samples. The Student’ *t*-test (*, *p* < 0.05) was used to statistically compare the gene expression profiles in *M. truncatula* seeds, where HP2 and HP4 were reported to the dry seeds (DS) for each gene.

Principal component analysis (PCA) was performed using all measured parameters (germination %, T_50_, seedling length, FW, DW, gene expression profiles) and the ClustVis program available at https://biit.cs.ut.ee/clustvis/ [36].

## 3. Results

### 3.1. Hydropriming Enhances Seed Germination

**Figure 2:**
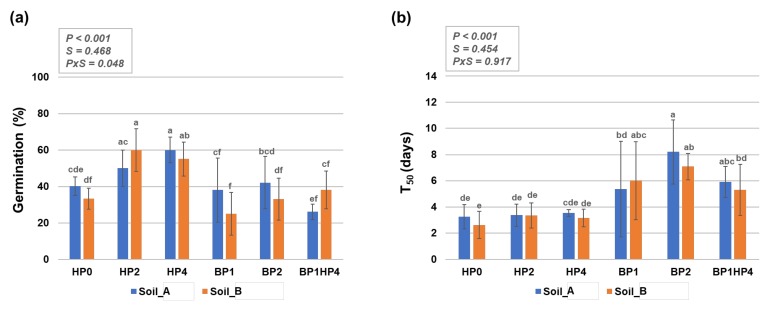
Germination of primed and non-primed *Medicago truncatula* seeds grown on two types of agricultural soils (Soil_A, TDS 82.9; Soil_B, TDS 103) collected from Northern India (Karsara, Varanasi). (**a**) Germination percentage (%). (**b**) The time required for 50% of seeds to germinate (T_50_). Data are represented as means ± SD of five replicates. Significant differences are shown with lowercase letters and the *P*-values are included in the upper panel of each graphic (P, priming; S, soil, PxS, interaction between priming and soil). HP0, non-primed control; HP2, 2 h hydropriming; HP4, 4 h hydropriming; BP1, *Bacillus* strain isolated from mustard rhizosphere; BP2, *Bacillus* strain isolated from and linseed rhizosphere; BP1HP4, 4 h of hydropriming and BP1 strain.

In non-primed seeds (HP0), the germination percentage on Soil_A was 40.2 ± 5.1% while a slight decrease (33.2 ± 5.8%) was observed when seeds were grown on Soil_B where higher TDS values were measured (Figure 2a). The relatively low basal germination in soil may be due to the higher than optimal temperatures registered at the growing site; the optimal temperature for *M. truncatula* seed germination was reported to be around 18–20 °C [37]. Hydropriming had significantly increased seed germination percentage on both types of soil. The highest germination percentage was observed with the HP2 treatment (60 ± 11.7%) on Soil_B and HP4 treatment (60 ± 9.6%) on Soil_A, respectively. Conversely, biopriming and the combination between biopriming and hydropriming did not result in any significant differences compared to non-primed samples (HP0) (Figure 2a). The overall differences between the imposed conditions, from a statistical point of view, are mostly due to the priming treatments (*p* < 0.001) rather than the soil type (*S* = 0.468).

When considering the germination speed (T_50_), it is possible to observe that non-primed seeds germinated in about 3 days, independently of the type of soil (Figure 2b). In this case, the hydropriming treatments did not result in any significant differences compared to the non-primed control. However, biopriming seemed to negatively affect germination speed as the maximum percentage of germination was reached in up to 8 days in some of these cases (e.g., BP2). The high standard deviations are because the germination of these seeds was not uniform. When addressing the uniformity of seed germination, an important aspect to be considered is the degree of seed dormancy. *Medicago truncatula* seeds show both physical and physiological dormancy [38]. Physical dormancy (the inability of water to penetrate the seed) is widely encountered in legume species whereas, in *M. truncatula*, physiological dormancy is generally removed during after-ripening [39]. A more recent study reports that freshly harvested seeds are most dormant at temperatures above 17 °C, whereas after-ripening increases the temperature limits for germination [40]. The seed lot used in the current study was harvested after-ripening in 2016, hence the temperature ranges for germination are not restricted to 17–20 °C. Moreover, the hydropriming treatments can be looked upon as a modality to break the physical dormancy. This is also in agreement with the fact that hydroprimed seeds germinated faster and more uniformly than the bioprimed seeds.

### 3.2. Hydropriming and Biopriming Treatments Improve Seedling Biomass

**Figure 3:**
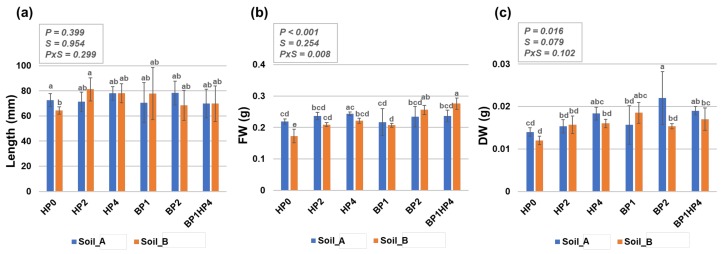
*Medicago truncatula* seedling growth on two types of agricultural soils (Soil_A, TDS 82.9; Soil_B, TDS 103) collected from Northern India (Karsara, Varanasi). (**a**) Seedling length (mm). (**b**) Measurement of fresh weight (FW, g). (**c**) Measurement of dry weight (DW, g). Data are represented as means ± SD of five replicates collected at 14-days after sowing. Significant differences are shown with lowercase letters and the *P*-values are included in the upper panel of each graphic (P, priming; S, soil, PxS, interaction between priming and soil). HP0, non-primed control; HP2, 2 h hydropriming; HP4, 4 h hydropriming; BP1, *Bacillus* strain isolated from mustard rhizosphere; BP2, *Bacillus* strain isolated from and linseed rhizosphere; BP1HP4, 4 h of hydropriming and BP1 strain.

If the germination percentage is more influenced by the hydropriming treatments, the seedling growth, on the other hand, seems to be more influenced by the biopriming treatments. This is mostly reflected by enhanced biomass rather than seedling length, as no statistically significant differences are observed in this parameter in most of the cases. However, a significant difference was observed when seedlings grown from non-primed seeds (HP0) on Soil_B as compared with the non-primed seeds grown in Soil_A; namely, a decrease in seedling length is noticed when seeds are grown in Soil_B (Figure 3a, HP0), characterized by higher TDS values. Nonetheless, HP2 treatment have rescued the seedling growth on Soil_B type as reflected by the significant difference between HP0 and HP2 in this soil. The fact that seedling growth is affected by the type of soil is evident also when measuring seedling biomass (reflected by the FW values) under non-primed conditions (HP0, Soil_A vs. Soil_B) (Figure 3b). Priming treatments resulted in significantly improved FW (HP0 vs. HP2, HP0 vs. HP4, HP0 vs. BP1, HP0 vs. BP2, and HP0 vs. HP4BP1) when seedlings were grown in the Soil_B (Figure 3b). Differently, no statistically significant differences were observed between primed and non-primed seedling for the FW parameter on Soil_A. When considering the DW parameter (Figure 3c), significantly higher values were registered only for the BP2 treatment on Soil_A (HP0 vs. BP2, Soil_A) and the BP1HP4 treatment on Soil_B (HP0 vs. BP1HP4, Soil_B). From a statistical point of view, the broad differences between the observed responses are primarily due to the priming treatments (FW, *p* < 0.001; DW, *p* < 0.05), and the interaction between priming and soil type (PxS < 0.05) regarding the FW parameter.

### 3.3. Expression Patterns of Genes Involved in DNA Repair and Antioxidant Response

**Figure 4:**
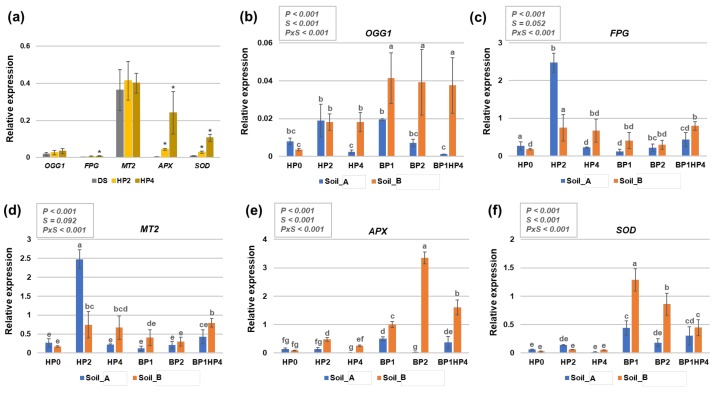
Gene expression patterns in *Medicago truncatula* seeds and seedlings grown from primed and non-primed seeds on two types of agricultural soils (Soil_A, TDS 82.9; Soil_B, TDS 103) collected from Northern India (Karsara, Varanasi). (**a**) Relative expression of *OGG1*, *FPG*, *MT2*, *APX*, and *SOD* genes in dry seeds (DS) and seeds treated with hydropriming for 2 h (HP2) and 4 h (HP4). Asterisks ‘*’ show statistical significance (*p* < 0.05) compared to dry seeds (DS). (**b**) Relative expression of *OGG1* gene in 14-days-old seedlings. (**c**) Relative expression of *FPG* gene in 14-days-old seedlings. (**d**) Relative expression of *MT2* gene in 14-days-old seedlings. (**e**) Relative expression of *APX* gene in 14-days-old seedlings. (**f**) Relative expression of *SOD* gene in 14-days-old seedlings. Data are represented as means ± SD of five replicates. Significant differences are shown with lowercase letters and the *P*-values are included in the upper panel of each graphic (P, priming; S, soil, PxS, interaction between priming and soil). HP0, non-primed control; HP2, 2 h hydropriming; HP4, 4 h hydropriming; BP1, *Bacillus* strain isolated from mustard rhizosphere; BP2, *Bacillus* strain isolated from and linseed rhizosphere; BP1HP4, 4 h of hydropriming and BP1 strain.

In our previous works, we reported that genes playing roles in DNA repair and antioxidant responses can be used as indicators of seed quality and seeding growth [5,6,7,8]. Here we focused on the expression patters of *OGG1* (8-Oxoguanine DNA glycosylase) and *FPG* (Formamidopyrimidine-DNA glycosylase), involved in Base Excision Repair (BER), *APX* (Ascorbate peroxidase) and *SOD* (Superoxide dismutase), encoding known ROS scavengers, and *MT2* (Metallothionein type 2), proven to act both as ROS scavenger and repair enzyme [41]. The expression patterns of these genes were tested both in dry and hydroprimed seeds (Figure 4a) as well as in the two-weeks-old seedlings grown in soil (Figure 4b–f).

In seeds, it is possible to observe that 2 h and 4 h of hydropriming did not significantly change the relative expression of *OGG1* and *MT2* genes as compared to dry seeds (DS). Conversely, significant upregulation was registered for the *SOD* and *APX* genes in response to both hydropriming timepoints while the FPG gene was upregulated only in response to the HP4 treatment (Figure 4a).

When considering the *OGG1* gene expression, it is possible to observe that the seedlings grown from hydroprimed and bioprimed seeds in Soil_B presented a significant upregulation of the OGG1 gene compared to HP0 (Soil_B) (Figure 4b). As for the FPG, downregulation of the gene is observed in non-primed seeds grown in Soil_B compared with Soil_A (HP0, Soil_A vs. Soil_B) (Figure 4c). The hydropriming treatment for 2 h (HP2) resulted in a significant upregulation of the gene in both Soil_A and Soil_B, compared to HP0 of each soil type. A similar trend was observed in the case of the MT2 gene, where hydropriming (HP2) and the combination between hydropriming and biopriming (BP1HP4) resulted in enhanced gene expression compared to HP0 (Figure 4d). Differently, the expression of *APX* and *SOD* genes was more influenced by the biopriming treatments as well as the BP1HP4 combination. In the case of the *APX* gene, the highest expression was induced by the BP2 treatment on Soil_B (Figure 4e) whereas the *SOD* gene peaked when the BP1 treatment was implemented on the same soil type (Figure 4f).

### 3.4. Integrative Data Analysis

**Figure 5:**
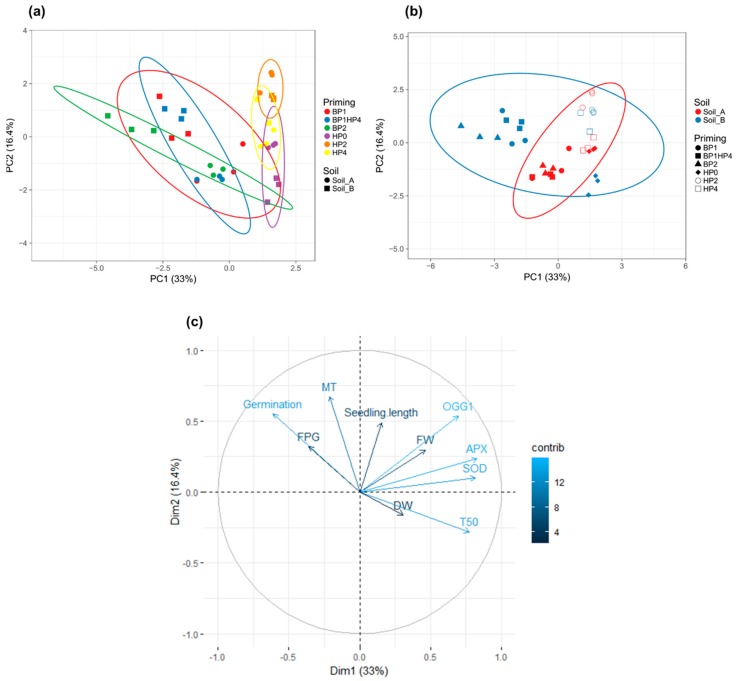
Principal component analysis (PCA). (**a**) Loading plot explaining the distribution of samples focusing on priming treatments. (**b**) Loading plot explaining the distribution of samples focusing on soil type. (**c**) Loading plot explaining the contribution of each measured variable (germination, T50, seedling length, FW, DW, SOD, APX, OGG1, MT, FPG). HP0, non-primed control; HP2, 2h hydropriming; HP4, 4 h hydropriming; BP1, *Bacillus* strain isolated from mustard rhizosphere; BP2, *Bacillus* strain isolated from and linseed rhizosphere; BP1HP4, 4 h of hydropriming and BP1 strain.

To better understand how the samples behave comparatively, a PCA analysis was performed taking into consideration the imposed conditions (soil type and priming treatments) and measured variables (germination %, T_50_, seedling length, FW, DW, and gene expression profiles) (Figure 5). The two main principal components extracted accounted for 49.4% of the variance. Unit variance scaling is applied to rows; SVD (singular value decomposition) with imputation is used to calculate principal components. X and Y axis show principal component 1 (PC1) and principal component 2 (PC2) that explain 33% and 16.4% of the total variance, respectively. Prediction ellipses are such that with probability 0.95, a new observation from the same group will fall inside the ellipse. In order to better understand the possible behaviors due to the priming treatments or soil type, two distinct PCA loding plots were constructed. In the first one (Figure 5a), the prediction elipses were drawn with a main focus on priming treatments whereas in the second one (Figure 5b) the focus is on soil type. It is thus possible to notice that the priming treatments, rather than soil type, have a higher influence on samples behaviour. This is in agreement with the statistical analyses sowing that the differences between samples are mainly due to priming treatments. When considering the contribution of each measured variable, it is possible to distinguish that germination percentage, T_50_, and MT2, OGG1, APX, and SOD gene expression patterns (light blue) had the most significant impact on the imposed treatments (Figure 5c).

## 4. Discussion

Agriculture represents a major stamina of the economic and socio-political stability of India and employs the largest workforce. About 60% of the geographical area of the country is occupied by agricultural land, most of which are facing one or more types of soil deterioration [42]. Legumes can help restore soil organic matter when used in rotation with non-leguminous crops. Thus, improving legume seed germination using easy and economically accessible methods will upgrade the livelihood of many small farmers.

Within this context, the present study focused on the evaluation of hydropriming and biopriming treatments as a means to improve seed germination and seedling establishment on agricultural soils collected from two sites in Northern India. The collected soils originate from the village of Karsara, where a power plant is located and dedicated to convert the municipal solid waste to electric power in an attempt to dispose waste through a sustainable and environmentally friendly process. However, the farmers in the region report loss of agricultural productivity and former agricultural soils were left barren. Measurements of basic soil physicochemical properties, such as pH, electrical conductivity (EC), or the content of total dissolved solids (TDS), are still looked upon as useful indicators of soil quality [43]. In the current experimental design, the measured TDS values were shown to be higher in the soils collected closer to the power plant (Soil_B, Table 1). TDS also refers to the total amount of soluble salts (expressed in parts per million, ppm) present in a soil, and it is perceived as an indicator of salinity problems. In accordance, EC represents the measure of electrical conductivity and is also indicative of soil salinity, saturation percentage, and water content [44]. Correlations between EC and forage legume productivity were studied and a nonlinear relationship was evidenced [45]. It was shown that forage legumes could tolerate a certain range of EC, where optimal values were between 0.15–0.22 ds/m and the maximal ones (where loss of productivity was much evidenced) reached to 0.44 ds/m [45]. Based on these parameters, *Medicago* forage species are considered to have a moderate-to-high tolerance to salinity conditions. Another indicative soil parameter measured in this study is the pH. Based on the performed measurements, the soils seem to be moderately alkaline (defined range 7.9–8.4), probably due to the presence of carbonates (e.g., sodium bicarbonate). As indicated by the Cornell University Cooperative Extension, the normal growth pH range for legumes is reported to be around 6.5–7.5 while the recommended range is limited to 6.6–7.0 [46]. Soil pH greater than 8.5 may indicate the presence of high amounts of K, Ca, and Mg, which may interact with phosphorus (P) and reduce the availability of soil micronutrients to cause productivity losses [47]. Surely, a more detailed characterization of these types of soils may provide interesting clues about the possible types of contaminants present in the area, and would be also helpful to propose defined phytoremediation techniques. However, this will be the objective of more dedicated collaborative projects while this pilot study focuses mainly on the influence that priming techniques could have on forage legumes seed germination in soil rather than under lab conditions.

Considering the priming techniques used in this study, hydropriming was chosen mainly because it is very easy to implement, and hence highly accessible also to farmers from underdeveloped regions. The biopriming formulations were selected from local varieties of rhizospheric microorganisms having plant growth-promoting traits under stressful conditions and studied in relation to improved phosphorus nutrition [48,49]. Moreover, biopriming can be associated with the elicitation of plant immunity, starting from the seedling stage [50]. The experiment was setup under the natural weather conditions of Varanasi to evaluate the efficiency of the tested protocols in the region. Our results showed that hydropriming treatments resulted in better germination on both types of soils (Figure 2). This is in agreement with other literature showing that hydropriming mainly improves germination as a result of the enhanced water uptake and more favorable water relations in primed seeds [51]. Additional reports support the positive effects of hydropriming in legumes by showing that this treatment was able to mitigate the negative effects of cold temperatures on the germination and seedling development in narrow-leaf lupine (*Lupine angustifolius*) [15]. On the other hand, biopriming had a more positive effect during seedling growth, as evidenced by the observed enhanced biomass (Figure 3). The biopriming treatments affect the germination process at later stages, for instance when the radicle protrudes and it becomes directly exposed to bacteria that can aid in soil nutrient uptake [17,49].

The next step was to examine the expression patterns of genes that play roles in DNA repair and antioxidant defense, previously used as indicators of seed quality during the early stages of germination (namely seed imbibition) [5,6,7,8]. This is a rather important aspect because such molecular indicators allow the prediction of seed quality in a very short time, thus providing assistance to Seed Companies to design customized priming protocols [2,3,9,10]. The qRT-PCR analyses shown that the *FPG, APX,* and *SOD* genes were upregulated in hydroprimed seeds while the *OGG1* and *MT2* genes were not responsive at the tested time points (Figure 4a). Nonetheless, in the 14-days-old seedlings, all the tested genes were differentially expressed in response to both priming and soil conditions. The *OGG1* and *FPG* genes play similar roles in BER pathway being involved in the removal of oxidized bases like 7,8-dihydro-8-oxoguanine (8-oxo-dG) and formamidopyrimidine (FAPy) lesions [29]. These had been previously reported to be upregulated during seed imbibition under physiological conditions or in the presence of osmotic stress (polyethylene glycol, PEG) in *M. truncatula;* their expression was mostly induced after 8 h of imbibition with water and 12 h of imbibition in the presence of PEG [29]. Other treatments with histone deacetylase inhibitors, for example trichostatin A (TSA) and sodium butyrate (NaB), have confirmed the enhanced expression of these genes during the first stages of seed germination [6,7]. Here, we show the upregulation of *OGG1* and *FPG* genes in response both hydropriming and biopriming (Figure 4b,c). This indicates that primed seedlings are better equipped to repair the DNA damage that can be induced by the possible presence of contaminants in the soil. Similarly, the *APX* and *SOD* genes, involved in the antioxidant response, were upregulated in hydroprimed seeds. In *M. truncatula*, upregulation of *SOD* and *APX* genes has been previously reported starting from the 4th h of seed imbibition with water [5]. In seedlings, the genes were mostly upregulated in response to biopriming (Figure 4e,f). The SOD enzyme catalyzes the dismutation of superoxide anion to hydrogen peroxide (H_2_O_2_), while APX plays a key role catalyzing the conversion of H_2_O_2_ into water, using ascorbate as a specific electron donor [52,53]. Enhanced accumulation of antioxidant enzymes following different seed priming methods (e.g., osmopriming, halopriming, hormopriming) has been previously reported in a number of plant species (*Moringa oleifera* [54], *Vigna radiata* [55], *Triticum aestivum* [56], *Oryza sativa* [57], *Sorghum bicolor* [58], *Brassica napus* [59]), and these were even correlated with changes in gene expression patterns [58,59]. Another group of interesting ROS scavengers includes metallothioneins (MTs), small cysteine-rich proteins that accumulate in response to toxic levels of heavy metals [60]. Besides their role in heavy metal detoxification, MTs are part of the signaling pathway activated by nitric oxide [41,61] and have been associated with protection against oxidative injuries also at the nuclear level [62,63]. Under our experimental conditions, *MT2* seems to be mostly upregulated in hydroprimed seedlings (Figure 4d) while no changes in gene expression level were observed in the bioprimed seeds (Figure 4a). As in the case of *OGG1*, also the *MT2* gene was previously shown to be upregulated only later on during seed imbibition in *M. truncatula* [6]. In a different work, the *MT* gene expression was monitored in artificially aged seeds of two *Silene* species that differ in seed longevity and grow in contrasting habitats [64]. Based on several measured parameters (e.g., ROS accumulation, antioxidant potential, telomere length) and the differential expression of *MT* and *SOD* genes in aged seeds it was possible to distinguish between short- and long-lived seeds under seed bank storage conditions [65]. In a context where seed researchers are continuously facing the complexity of efficient seed germination and healthy seedling establishment, the genes hereby investigated (*OGG1, FPG, APX, SOD,* and *MT2*) are important molecular indicators to test and monitor seed quality and seedling development.

In conclusion, the present study shows that seed priming is an efficient method to enhance seed germination and seedling establishment in agricultural soils collected from abandoned agricultural areas of Northern India. While hydropriming improved seed germination percentage, biopriming resulted in improved seedling development. At a molecular level, this is reflected by upregulation of specific genes used as molecular indicators of seed quality. The data hereby presented set the stage for future experimental designs where a more diverse range of crop species will be tested to develop ad-hoc priming protocols to boost the agricultural productivity under adverse environmental conditions in a more sustainable manner. Moreover, future studies could be tailored to include these germination enhancing techniques and different legume species to promote phytoremediation of degraded soils.

## Figures and Tables

**Table 1:** Measurement of soil parameters. Soil_A, Soil_B, types of soils collected from the indicated sites. EC, electric conductivity; TDS, content of total dissolved solids.

Sample	pH	EC (ds/m)	Salinity (ppt)	TDS (PPM)
Soil_A	8.1	0.15	0.07	82.9
Soil_B	8.23	0.2	0.09	103

## Supplementary Materials

The following are available online at https://www.mdpi.com/2073-4425/11/3/242/s1, Figure S1. geNorm analysis of reference genes. Table S1. List of oligonucleotides used for the qRT-PCR analysis.
