# Supplementary material for: Hydropriming and Biopriming Improve Medicago truncatula Seed Germination and Upregulate DNA Repair and Antioxidant Genes
Source: Genes (Basel). 2020 Feb 25;11(3):242. doi: 10.3390/genes11030242 (PMC7140799; doi:10.3390/genes11030242)
Supplement: Supplementary file 1 [file genes-11-00242-s001.pdf]

genes

Supplementary Material

Hydropriming and biopriming improve *Medicago truncatula* seed germination and upregulate DNA repair and antioxidant genes

Chiara Forti[1], Ajay Shankar[2], Anjali Singh[2], Alma Balestrazzi[1], Vishal Prasad[2] and Anca Macovei[1,*]

[1] Department of Biology and Biotechnology 'L. Spallanzani', University of Pavia, via Ferrata 9, 27100-Pavia, Italy;
[2] Institute of Environment and Sustainable Development, Banaras Hindu University, Varanasi 221005, India;
* Correspondence: anca.macovei@unipv.it; Tel.: +39-0382-985583

Table 1. List of oligonucleotides used for the *q*RT-PCR analysis. For each gene the Phytozome (https://phytozome.jgi.doe.gov/pz/portal.html) repository accession is indicated. The oligonucleotides were designed using Primer3 (http://primer3.ut.ee/) and subsequently checked for sequence specificity with NCBI BLAST (https://blast.ncbi.nlm.nih.gov/Blast.cgi?PAGE_TYPE=BlastSearch) while primers physical characteristics were analysed using IDT OligoAnalyzer Tool (https://eu.idtdna.com/pages/tools/oligoanalyzer).

Gene	Accession	Forward primer (5′-3′)	Reverse primer (5′-3′)	Efficiency
OGG1	Medtr3g088510	AAACACCGCACCTTCTCAAT	TGTGGAGATGTTTGAGGGAA	1.73
FPG	Medtr2g126800	TCCTTTCAATTCGGTATGGC	GCTCCAAACCATCGTCTAGC	1.76
SOD	Medtr7g114240	CCTGAGGATGAGACTCGACA	GAACAACAACAGCCCTTCCT	1.79
APX	Medtr4g061140	AGCTCAGAGGTTTCATCGCT	CGAAAGGACCACCAGTCTTT	1.76
MT2	Medtr8g060850	CATGTCAAGCTCATGCGGCAAC	TGCCGTAGTTGTTTCCCTTCCC	1.72
ACT	Medtr3g095530	TCAATGTGCCTGCCATGTATG	ACTCACACCGTCACCAGAATC	1.70
TUB	Medtr7g089120	TTTGCTCCTCTTACATCCCGTG	GCAGCACACATCATGTTTTTGG	1.82
UBI	Medtr3g091400	GCAGATAGACACGCTGGGA	AACTCTTGGGCAGGCAATAA	1.81
GADPH	Medtr3g085850	TGCCTACCGTCGATGTTTCAGT	TTGCCCTCTGATTCCTCCTTG	1.75
ELF1α	Medtr6g021800	GACAAGCGTGTGATCGAGAGA	TTTCACGCTCAGCCTTAAGCT	1.69

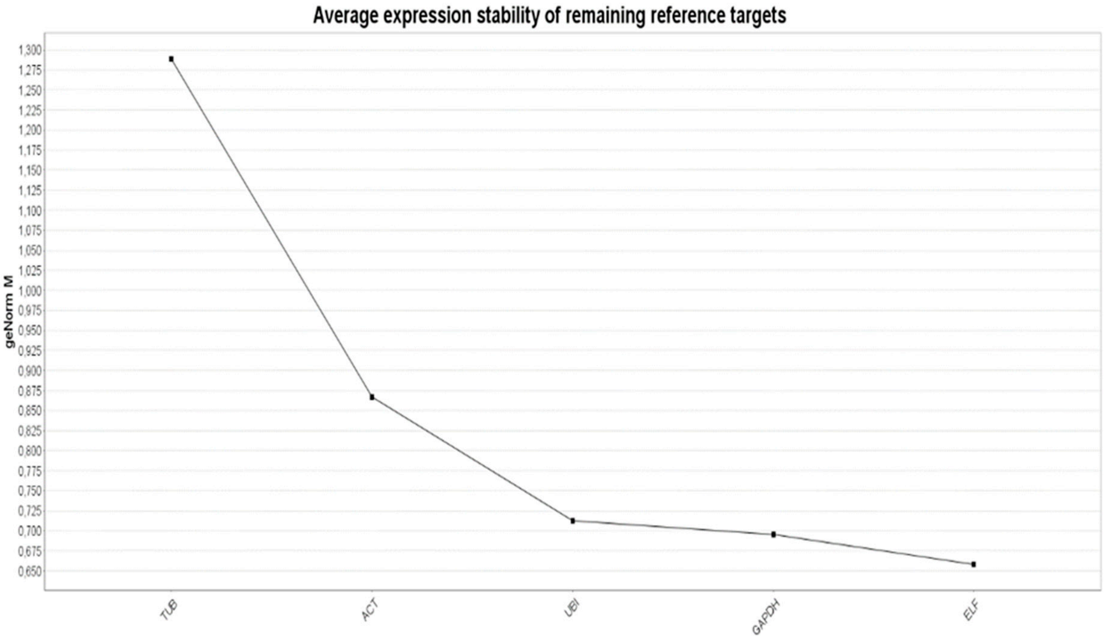

Figure 1. geNorm analysis of reference genes. geNorm is a popular algorithm (https://genorm.cmgg.be/) used to determine the most stable reference genes from a set of tested candidate reference. Here, we used the tubulin (*TUB*), actin (*ACT*), ubiquitin (*UBI*), glyceraldehyde-3-phosphate dehydrogenase (*GAPDH*) and elongation factor 1α (*ELF*) genes. The gene expression normalization factor (geNorm M) was calculated for each sample based on the geometric mean of the reference genes. The cDNA extracted from seedlings grown from primed and non-primed seeds was used for this analysis. The most stable genes, presenting the lowest geNorm M, were identified as *GADPH* and *ELF*, and were subsequently used for *q*RT-PCR data normalization, as indicated in Materials & Methods.
